# Cardiac Troponin Levels in Patients with Chronic Kidney Disease: “Markers of High Risk or Just Noise’’?

**DOI:** 10.3390/diagnostics14202316

**Published:** 2024-10-18

**Authors:** Eleni V. Geladari, Natalia G. Vallianou, Angelos Evangelopoulos, Petros Koufopoulos, Fotis Panagopoulos, Evangelia Margellou, Maria Dalamaga, Vassilios Sevastianos, Charalampia V. Geladari

**Affiliations:** 1 Department of Internal Medicine, Evangelismos General Hospital, 45–47 Ipsilantou Str., 10676 Athens, Greece; evmargellou@yahoo.gr (E.M.); vsevastianos@gmail.com (V.S.); 2 First Department of Internal Medicine, Sismanogleio General Hospital, 37 Sismanogliou Str., 15126 Athens, Greece; natalia.vallianou@hotmail.com (N.G.V.); peterkouf13@gmail.com (P.K.); fotis_1992@hotmail.com (F.P.); 3 Roche Diagnostics Hellas S.A., 4 Alamanas & Delfon Str., 15125 Athens, Greece; angelos.evangelopoulos@roche.com; 4 Department of Biological Chemistry, National and Kapodistrian University of Athens, 75 Mikras Asias Str., 11527 Athens, Greece; madalamaga@med.uoa.gr; 5 Hypertension and Cardiovascular Disease Prevention Center, Evangelismos General Hospital, 45–47 Ipsilantou Str., 10676 Athens, Greece; cgeladari@gmail.com

**Keywords:** troponin, high-sensitivity cardiac troponin, chronic kidney disease, cardiovascular risk, biomarkers

## Abstract

Kidney disease is linked to the development of cardiovascular disorders, further increasing morbidity and mortality in this high-risk population. Thus, early detection of myocardial damage is imperative in order to prevent devastating cardiovascular complications within this patient group. Over the years, cardiac biomarkers have been identified and are now widely used in everyday clinical practice. More specifically, available data suggest that cardiac troponin and its regulatory subunits (TnT, TnI, and TnC) reflect the injury and necrosis of myocardial tissue. While cTnC is identical in cardiac and skeletal muscle, TnT and TnI constitute cardiac-specific forms of troponin, and, as such, they have been established by international societies as biomarkers of cardiac damage and diagnostic indicators for acute myocardial infarction. Elevations in the levels of both cardiac troponins (cTnT and cTnI) have been also reported in asymptomatic patients suffering from chronic kidney disease. Therefore, if abnormal, they often generate confusion among clinicians regarding the interpretation and clinical significance of their numerical values in emergency settings. The aim of this review is to explore the reasons behind elevated troponin levels in patients with chronic kidney disease and identify when these elevated levels of biomarkers indicate the need for urgent intervention, considering the high cardiovascular risk in this patient group.

## 1. Introduction

It is widely known that patients with chronic kidney disease (CKD) have an increased risk of suffering cardiovascular events. According to the National Institute of Diabetes and Digestive and Kidney Diseases (NIDDKD), the prevalence of cardiovascular disease (CVD) is 69.6% among CKD patients >65 years old, which can be compared to 34.7% for individuals with normal kidney function [1]. Previous studies have established that there is a graded association between a reduced estimated glomerular filtration rate (eGFR) and the risk of CVD events, including acute myocardial infarction (AMI) [2,3]. Angiographic assessment has demonstrated that up to 40–50% of incident dialysis patients have significant coronary artery disease (CAD) [4,5]. Indeed, an AMI is often a lethal event in patients with CKD; two-year post-AMI mortality rates have been reported to escalate up to 73% among patients with end-stage renal disease (ESRD) [6].

CVD accounts for more than half of all deaths among patients with ESRD. Arrhythmias and cardiac arrest alone are responsible for more than one-third (37%) of CVD deaths. Atherosclerotic heart disease along with left-ventricular hypertrophy (LVH) are the most common manifestations that link CVD to CKD. Interestingly, CVD begins early in the course of CKD. The more advanced the stage of CKD, the more prevalent the CVD burden. Unfortunately, complete and appropriate testing to evaluate cardiac function is not offered to all CKD patients, and thus many CVD complications are discovered when patients are already in moderate to severe stages of CKD [1,7]. Hence, it is of utmost importance to detect CVD damage as early as possible in the course of renal disease in order to mitigate detrimental outcomes, provide better quality of care, and prolong patient survival [1].

In the context of suspected cardiac events among patients with CKD, cardiac biomarkers constitute a useful tool for diagnosing AMI or stable ischemic heart disease (SIHD). Currently, it is known that cardiac troponins are components of the contractile apparatus of myocardial cells that reflect injury leading to the necrosis of myocardial cells [8]. Besides identifying myocardial necrosis, these biomarkers help clinicians determine cardiac systolic dysfunction, LVH, and coronary artery disease (CAD) even in subjects with ESRD [8,9,10]. However, the diagnostic ability and prognostic value of cardiac troponin T (cTnT) and cardiac troponin I (cTnI) in patients with CKD are uncertain in the emergency setting. Elevations in the levels of both cardiac troponins (cTnT and cTnI) have been reported for asymptomatic patients with CKD [11]. As a result, these elevations have often been described as non-specific for myocardial injury and, therefore, disregarded by clinicians [12].

Yet, in the clinical setting, this is an essential problem among CKD patients since there are no reference ranges for cardiac troponin that take into account, at least in part, kidney function. In one study, nearly 30% of asymptomatic dialysis patients showed cTnT levels above the cutoff value of 100 ng/L for myocardial damage [13]. This is thought to be one of the reasons why cTnT is not used for diagnosing acute coronary events in patients with renal insufficiency. Therefore, two major questions arise: Can clinicians always be confident that elevated levels of troponins in CKD patients can be ignored? Is there any threshold or are there any additional signs and symptoms that raise a high clinical suspicion of an ongoing acute coronary syndrome (ACS)?

Understanding the clinical significance of elevated cTnT and cTnI levels in this patient population is extremely important as individuals with CKD have a higher pretest likelihood of having ACS [14,15]. The purpose of this review is to clarify why CKD is often accompanied by elevated troponin levels as well as when this should be a warning sign indicating the need for an emergency intervention. Apart from acute clinical presentations, we will elaborate upon the significance of elevated cardiac troponin levels among CKD patients as a marker of CAD in the long run.

## 2. Characteristics of Troponins: Structure and Functions

**Figure 1:**
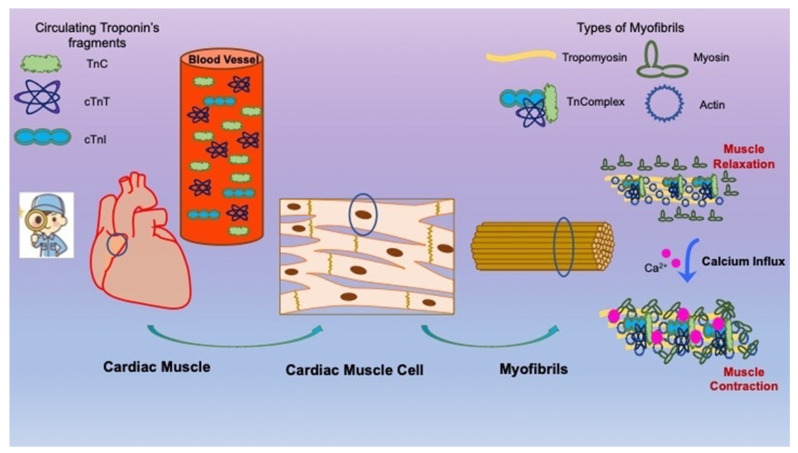
The cardiac muscle depends on the cardiac muscle cells for their contraction. Myofibrils, such as tropomyosin, troponin complex, myosin, and actin, all counteract when there is an influx of calcium into the myoblast to induce myoblast contraction. When necrosis of cardiomyocytes occurs, especially involving a large number of cardiac muscle cells, there is release of troponins in the blood circulation, accounting for a significant rise in the blood levels of troponins. This absolute increase in the blood levels of cTnI and cTnT may translate into the use of cTnI and cTnT as biomarkers of myocardial damage.

Troponin is a protein complex consisting of three regulatory subunits: Troponin T (TnT), Troponin I (TnI), and Troponin C (TnC). Troponin plays a crucial role in the contractile process of skeletal and cardiac muscle, whereas smooth muscles lack troponin. Troponin is bound to another protein called tropomyosin. In the relaxed cardiac muscle, it blocks the binding of myosin to actin filaments. In sharp contrast, when calcium ions influx into the cardiac sarcoplasm, they bind to the troponin complex, causing a conformational change, which allows for the binding of myosin to actin filaments, resulting in cardiac muscle contraction [16,17,18]. Approximately 90% of troponin is found bound to the other myofilaments, while around 10% remains protein-free in the cytosol. This free cytosolic troponin is augmented in cases of muscle injury, wherein leakage from the damaged muscle membrane occurs. Thus, the augmentation of the free cytosolic troponin is the result of muscle injury or death, leading to increases in serum troponin levels [16,17,18]. Troponin C forms are identical in cardiac and skeletal muscle, while there are cardiac-specific forms of Troponin I and T, namely, cTnI and cTnT (Figure 1).

Due to this cardiac specificity, cTnI and cTnT are currently recommended for use as biomarkers of cardiac damage and diagnostic indicators for AMI [8,16,17,18].

## 3. Assays for Cardiac Troponins

Notably, numerous assays with variable sensitivity and specificity regarding cTnT and cTnI in patients with ACS have been introduced [19,20,21,22]. The most recent assays for cardiac troponin detection are characterized by excellent sensitivity, which comes with minor only compromises in specificity. Hence, although the probability of a false-positive diagnosis of an ACS is higher than it would be with less sensitive assays, the number of potentially missed diagnoses is minimal [23,24,25,26]. Therefore, these markers constitute powerful tools for risk stratification and therapeutic decision-making in regard to patients with non-ST-segment elevation AMI (NSTEMI) [23]. More specifically, the first 5th-generation high-sensitivity cTn (hs-cTn) was instituted in 2010, and it gained approval by the Food and Drug Administration (FDA) in 2017. The coefficient of variation (CV%) is a standardized measure of the dispersion of a probability distribution. It is used to describe precision and repeatability in analytical chemistry [17]. In general, the smaller the CV%, the better the precision of the assay. Fifth-generation assays for hsTnT and hs-TnI were characterized by a CV% of <10 for the 99th-percentile upper reference limit (URL), whereas 4th-generation assays for cardiac troponins had a CV between 10–20% at the 99th-percentile URL [19].

Friden et al. have demonstrated in animal models as well as in human beings that under normal circumstances, i.e., when no myocardial infarction is present, the extent of the renal clearance of cardiac troponins is relatively small. On the contrary, in cases of high serum levels of cardiac troponins, as in AMI, extrarenal clearance seems to predominate [20]. This remarkable finding may account for the mainly renal clearance of cardiac troponins in low serum concentrations of cTn, which can be measured using an hs-cTn assay and are not measurable with previous-generation cTn assays [20].

## 4. Cardiac Troponins in CKD

### 4.1. AMI and CKD

According to the Fourth Universal Definition of MI (UDMI), MI is defined as the presence of cTn > 99th percentile of URL in the clinical setting of myocardial ischemia [27]. More specifically, AMI is defined as an acute rise and/or fall in cTn of at least 1 value >99th percentile of URL accompanied by symptoms of acute cardiac ischemia, ECG changes, and/or imaging evidence consistent with myocardial ischemia [28,29]. The 2021 AHA/ACC (American Heart Association/American College of Cardiology) guidelines point out the significance of hs-cTn, the 99th URL, and the clinical symptoms and signs. In addition, these guidelines have underlined the sex differences as well as differences in other subpopulations, such as patients with CKD [30]. In particular, women who present to the ED (Emergency Department) with chest pain are more frequently underdiagnosed, and cardiac causes should not be overlooked. Moreover, women tend to have other relevant comorbidities, such as dyslipidemia and hypertension, and they also more often present with atypical symptoms, i.e., palpitations or jaw/back pain, when compared to men [30]. Therefore, the 2021 AHA/ACC guidelines for the evaluation and diagnosis of chest pain strongly recommend special consideration regarding sex differences as well as differences in patients with CKD [30]. More specifically, there is accumulating data suggesting that women have a lower 99th-percentile URL than men [30,31]. Thus, hs-cTnT and hs-cTnI differential sex 99th percentiles are recommended [31]. For most hs-cTn assays, sex-specific-99th-percentile URLs are used, although there is no consensus on these sex-specific-99th-percentile URLs regarding all hs-cTn assays [30,31].

As already mentioned, patients with CKD, especially severe CKD, exhibit elevated levels of hs-cTn. These almost invariable increases in hs-cTn levels are suggested to be multifactorial. Parameters such as decreased renal clearance and various degrees of myocardial ischemia due to inflammation and uremic toxins together with anemia may be implicated [32,33,34]. It is noteworthy that among patients with severe CKD, patients who underwent revascularization for an AMI had significantly higher hs-cTn levels when compared to patients who did not undergo revascularization. In addition, among patients with ESRD, it was proposed that higher cut-off values for hs-cTnT should be used for diagnosing AMI, while in stage 3 CKD, physicians can follow lower hs-cTnT cut-off values for AMI [35]. Whether higher hs-cTn cut-off values for patients with CKD should be established remains controversial. It is possible that a higher hs-cTn cut-off value could be instituted for patients undergoing dialysis or patients with ESRD in general. However, among patients with CKD of stages 1, 2, and 3, there appears to be no statistically significant difference, and further large-scale studies are needed to shed light upon this debatable issue. Notably, the vast majority of studies regarding the diagnosis of AMI have been performed on patients with normal renal function, while there is a lack of large cohort studies concerning patients who present with chest pain and CKD. This lack of studies accounts for the paucity of data regarding hs-cTn cut-off values in this particular population [35,36,37].

Unlike the 2021 AHA/ACC guidelines, the 2023 ESC (European Society of Cardiology) guidelines endorse the rule in/rule out algorithm for diagnosing AMI [37]. In particular, 0 h/1 h and 0 h/2 h testing with hs-cTn have an NPV (negative predictive value) >99% for ruling out AMI and are very useful in the evaluation of chest pain in the ED [38]. It is noteworthy that different hs-cTn assays use different cut-off values in the 0 h/1 h and 0 h/2 h algorithm. Despite the fact that different hs-cTn assays have different cut-off values, the NPV of this algorithm has been assessed and well established in large cohort studies [37,38,39,40,41,42]. Furthermore, it is widely known that patients with CKD are more prone to ACS when compared to the general population [38,39,40,41,42,43]. However, among patients with CKD, hs-cTn levels may be increased due to the decreased renal clearance of hs-cTn. Therefore, serial hs-cTn level measurements are of the utmost importance for patients with CKD. A lack of increase in serial measurements of serum hs-cTn levels makes AMI highly unlikely. It is noteworthy that among patients with CKD, presentation with chest pain in the ED indicates the need for serial hs-cTn measurements, as CKD patients have a high pretest probability for AMI [38,39,40,41,42,43]. Twerenbold et al. have evaluated the rule-in/rule-out algorithm for AMI with respect to 3254 patients, 15% of whom (487 patients) had CKD. In this study population, the sensitivity of the rule-out algorithm for AMI was very high for both groups, i.e., patients with normal renal function and patients with CKD. More specifically, when using hs-cTnT, the sensitivity was 100% in the CKD group and 99.2% in the group with normal renal function, whereas the specificity for the rule-in algorithm was 88.7% in the CKD group and 96.5% among patients with normal renal function. The overall efficacy in regard to the CKD group was significantly lower when compared to that of the group with normal renal function (51% versus 81%, *p* < 0.001) [44]. Despite the fact that the sensitivity of hs-cTn levels for diagnosing AMI among patients with CKD remains very high, their specificity is lower due to the persistently increased levels of hs-cTn in this population. This drawback may be overcome through the performance of serial serum hs-cTn concentrations to exclude any pre-existing elevations, which could be suggestive of AMI. A carefully acquired medical history, any ECG changes, and imaging studies would be very helpful in this clinical setting. The GRACE (Global Registry of Acute Coronary Events) score is an old risk stratification score that includes serum creatinine levels and cardiac biomarkers, but not eGFR, among other relevant parameters [45]. However, the GRACE score was developed in 2003, prior to the initiation of the fifth-generation hs-cTn. The same applies for the TiMI (Thrombolysis in Myocardial Infarction), PURSUIT (Platelet Glycoprotein IIb/IIIa in Unstable Angina: Receptor Suppression Using Integrilin Therapy), and FRISK (Fast Revascularization in Instability in Coronary Disease) scores [46]. These three scores are also old and do not include the measurement of cardiac troponins, nor do they take into account eGFR or even serum creatinine levels [46]. The American College of Emergency Physicians endorses the HEART (history, ECG, age, risk factors, and troponin) score, with low scores classified as 0–3, moderate scores falling under 4–6, and high scores corresponding to 7–10. Based on these scores, the American College of Emergency Physicians suggests that a non-ischemic ECG together with negative hs-cTn levels at 0 h/2 h are predictive of a low rate of MACE (major adverse cardiovascular events) at 30 days [47,48,49,50]. In this regard, changing patterns or deltas of cTn are of paramount importance for serial measurements in terms of diagnosing AMI [51,52,53,54,55,56,57,58]. Deltas can be detected with higher precision than conventional cTn assays using hs-cTn assays [53,54,55,56,57,58,59,60,61]. However, there may be some imprecision regarding the detection of very small deltas due to several factors, mainly instrument-to-instrument variability and analytical outliers [53,54,55,56,57,58,59,60,61].

Only recently, Knott et al., in their observational study involving 1992 patients, amongst whom 25% (501 patients) had CKD, demonstrated that the diagnostic performance of the 0 h/2 h algorithm for ruling in AMI was inferior for patients with CKD [62]. A serum threshold of hs-cTnT at 0 h of >100 ng/L had a PPV (positive predictive value) of 53%, while a threshold of >300 ng/L had a PPV of 80%. Notably, the 2 h delta in serum hs-cTnT ≥10 ng/L was associated with a PPV of 66%, a delta of hs-cTnT >20 ng/L was associated with a PPV of 86%, and a delta of hs-cTnT >30 ng/L was associated with a PPV of 88% in the CKD subpopulation. Knott et al. concluded that for patients with CKD, the 0 h/2 h algorithm yielded an improved PPV when higher thresholds were used [62].

Notably, the 2023 ESC guidelines recommend that when the two measurements of hs-cTn in the rule-in/rule-out algorithm for AMI are inconclusive and no alternative diagnosis exists, a third measurement at 3 h should be performed [37]. In addition, no other cardiac biomarkers, such as CPK-MB or copeptin, should be used as adjuncts to hs-cTn for the diagnosis of AMI [37]. However, Chen et al., in their study, which included 5022 patients with AMI, among whom 797 patients (15.9%) had CKD, have shown that CPK-MB is an independent predictor of in-hospital mortality irrespective of renal function and reported that the ratio of hs-cTnT to CPK-MB may be helpful in the risk stratification of patients with AMI among patients with CKD [63].

Overall, serial measurements of hs-cTn are strongly recommended, especially among patients with CKD, as the detection of deltas has been documented to be very helpful in regard to this high-pretest-probability population [64]. Serial measurements among patients with CKD may have an extra cost but may prove to be life-saving and most probably cost-effective, as they could avoid unnecessary admissions to the hospital.

### 4.2. CAD and CKD

Cardiac troponins are released into the circulation following myocardial damage. Apart from ACS, SIHD has been associated with increases in serum hs-cTn levels among patients with CKD [65,66,67,68]. Oxidative stress, inflammation, and reductions in eGFR levels all contribute to the elevated hs-cTn levels in this population [32,33,34]. Nasr et al., in their study, enrolled 107 participants and demonstrated a correlation between serum hs-cTnT levels and echocardiographic parameters of left-ventricle (LV) dysfunction in patients with CKD when compared to healthy adults. They also found an association between increased serum levels of hs-cTnT and LVH (left-ventricular hypertrophy). Therefore, they concluded that hs-cTnT could be used as a very useful tool for the evaluation of cardiac structure and function among patients with CKD [68]. The same finding has also been reported in the KNOW-CKD study, which enrolled 2017 patients who had CKD of stages 1 to 5 but were not on dialysis. This cohort study also documented that serum levels of hs-cTnT correlate with echocardiographic parameters of LVH. More specifically, the KNOW-CKD Study has shown that increased hs-cTnT levels at baseline were predictive of the development of LVH after 4 years of follow up [69]. Sun et al. have also confirmed the usefulness of hs-cTnT together with NT-proBNP in terms of evaluating LVH and LV systolic as well as diastolic dysfunction [70].

Notably, Wada et al. studied the plausible association between high serum levels of hs-cTn and CVD events [71]. In particular, they enrolled 3255 patients with CAD, among whom 1301 (39.96%) had underlying CKD, and measured various biomarkers. Hs-cTnI was documented to be related to MACE, such as death from CVD, non-fatal MI, non-fatal stroke, hospitalization for heart failure, and revascularization (regarding the coronary or peripheral artery). They concluded that hs-cTnT together with NT-proPNP were the most powerful biomarkers for MACE [71]. Furthermore, Chesnaye et al. studied 176 patients aged >65 who had CKD of stages 4 and 5 but were not on dialysis, for whom information regarding hs-cTnT was available over time. In particular, they had a median of six measurements of cTnT per patient for a period of a median follow-up of 2.4 years. They reported that measurements of cTnT over time were associated with increased mortality risk. Based on their findings, they proposed that measurements of serum levels of cTnT could be a useful tool for the timely identification of older patients with CKD stages 4 and 5 who are at a higher mortality risk [72]. Furthermore, there is mounting evidence that the same applies for patients on dialysis as well [73,74,75,76,77,78]. Notably, Bangalore et al., in their study including 777 patients with advanced CKD (defined as eGFR levels <30 mL/min/1.73 m^2^ and SIHD), concluded that early invasive interventions, when compared to early conservative treatment, did not decrease the risk of death or non-fatal infarction. Moreover, they documented that patients who underwent early invasive interventions had a higher incidence of stroke, death, and initiation of dialysis [78].

## 5. Hs-cTn and Prediction of CVD Mortality in Patients with CKD

Various models are being explored regarding CVD mortality among patients with CKD. Mendonca et al. have recently proposed a four-variable model, comprising log-transformed NT-proBNP, log-transformed hs-cTnT, log-transformed albuminuria, and eGFR, for patients with CKD. The study enrolled 3718 patients from the Chronic Renal Insufficiency Control Study (CRIC), who were followed for a median of 9 years. They concluded that this four-variable score provided significant prognostic information regarding CVD death, hospitalization for heart failure, and all-cause mortality [79]. Matsushita et al. studied SCORE2 (Systematic Coronary Risk Evaluation 2) and SCORE2-OP (Systematic Coronary Risk Evaluation 2 in Older Persons) by adding CKD parameters for 5,997,719 individuals. In particular, they added eGFR alone, eGFR plus urinary ACR (albumin-to-creatinine ratio), and eGFR plus dipstick proteinuria [80]. They documented that these add-on scores for SCORE2 and SCORE2-OP, which had been integrated as a tool to predict increased risk for CVD within 10 years among apparently healthy individuals, improved CVD prediction for patients with CKD [81,82,83,84]. It seems likely that the addition of the above-mentioned CKD parameters to these two scores may contribute to improved CVD risk prediction for patients with CKD [82].

## 6. Increases in hs-cTn of NSTEMI (Non-ST Elevation Myocardial Infarction) Origin among Patients with CKD

Hs-cTn may also be increased due to various other causes, especially among patients with CKD. Table 1 depicts the major causes other than NSTEMI, which could result in serum hs-cTn elevations.

## 7. Conclusions

Undoubtedly, as hs-cTns were included in the definition of the Fourth UDMI as of 2018, they are still our best biomarker for the diagnosis of AMI. In addition, they have been beneficial in risk stratification among patients presenting to the ED with chest pain. Moreover, they seem to be of particular interest for predicting CVD risk among patients with CKD. Patients with stable coronary disease should adhere to non-pharmacological as well as pharmacological measures in order to improve their quality of life together with their life expectancy. Therefore, these patients’ timely identification is of the utmost importance and could be lifesaving. Despite the fact that hs-cTnT and hs-cTnI exhibit very high sensitivity and accuracy in diagnosing AMI, there is still room for improvement in the usage of these biomarkers in the clinical setting and in certain patient groups.

## Figures and Tables

**Table 1:** A summary of the major causes characterized by elevated serum hs-cTn levels other than AMI.

Myocardial Origin Causes	Non-Myocardial Origin Causes	Laboratory Causes
Inflammatory cardiac involvement, e.g., myocarditis, endocarditis, pericarditis	Pulmonary embolism	Hemolysis [84]
Cardiac tachyarrhythmias, e.g., atrial fibrillation	Other respiratory disorders, e.g., COPD, COVID-19, and other respiratory viruses	Heterophilic antibodies [84,85]
Cardiomyopathies, e.g., Takotsubo cardiomyopathy, hypertrophic cardiomyopathy, amyloidosis, sarcoidosis, etc.	Sepsis	Autoantibodies, RF [86]
Drugs, e.g., cocaine, methamphetamines, doxorubicin	CNS disorders, e.g., stroke, hemorrhage	Fibrin presence [84]
Cardiac interventions, such as cardiac bypass, ablation, PTCA	Severe anemia or other strenuous conditions, e.g., marathon, rhabdomyolysis, etc.	ALP [87,88]
Heart failure	AKI, CKD	Biotin [87,88]
Cardiac arrest	Shock	Macrotroponin [17]
Cardiopulmonary resuscitation	CO intoxication	[87,88]
Vasculitides with coronary involvement, e.g., SLE, Kawasaki		[87,88]
Aortic dissection		[87,88]
Severe hypertension		[88,89]

Abbreviations: AKI: acute kidney injury; ALP: alkaline phosphatase; CNS: central nervous system; CO: carbon monoxide; PTCA: percutaneous coronary angioplasty; RF: rheumatoid factor; SLE: systemic lupus erythematosus.

## Data Availability

No new data were created or analyzed in this study.

## List of Abbreviations

CKD: ACC: American College of Cardiology, ACS: acute coronary syndrome; AHA: American Heart Association; AMI: acute myocardial infarction; CAD: coronary artery disease; CKD: chronic kidney disease; cTn: cardiac troponin; CV: coefficient of variation; CVD: cardiovascular disease; ECG: electrocardiography; ED: emergency department; eGFR: estimated glomerular filtration rate; ESC: European Society of Cardiology; ESRD: end-stage renal disease; FDA: Food and Drug Administration; FRISK: Fast Revascularization in Instability in Coronary Disease; GRACE: Global Registry of Acute Coronary Events; HEART score: history, ECG, age, risk factor, and troponin score; LV: hs-cTn: high-sensitivity cardiac troponin; LV: left ventricular; LVH: left-ventricular hypertrophy; MACE: major adverse cardiovascular events; NIDDKD: National Institute of Diabetes and Digestive and Kidney Diseases; NSTEMI: non-ST elevation myocardial infarction; NPP: negative predictive value; NT-proBNP: N-terminal pro b-type natriuretic peptide; PPD: positive predictive value; PURSUIT: platelet glycoprotein IIb/IIIa in unstable angina: Receptor Suppression Using Integrilin Therapy; SIHD: stable ischemic heart disease; SCORE2: Systematic Coronary Risk Evaluation 2; SCORE2-OP: Systematic Coronary Risk Evaluation 2—Older Persons; TIMI: thrombolysis in myocardial infarction; UDMI: universal definition of myocardial infarction; URL: upper reference limit.
